# The impact of temperature and unwanted impurities on slow compression of ice†

**DOI:** 10.1039/d1cp03922a

**Published:** 2021-12-06

**Authors:** Christina M. Tonauer, Marion Bauer, Thomas Loerting

**Affiliations:** Institute of Physical Chemistry, University of Innsbruck, Innrain 52c Innsbruck A-6020 Austria thomas.loerting@uibk.ac.at

## Abstract

For slowly compressed hexagonal ice pressure-induced amorphisation to high-density amorphous ice (HDA) takes place below and at 130 K, but polymorphic transformation to ice IX takes place at 140–170 K. Stable ice II only forms above 170 K. Ice IX impurities trigger ice IX growth even at 120 K. HDA and ice IX are equally long-lived, where both can be regarded as metastable phases.

## Abstract 2

Slow compression of ice at 100 K usually results in pressure-amorphisation, unless there are ice IX seeds and temperature gradients.

## Graphical abstract

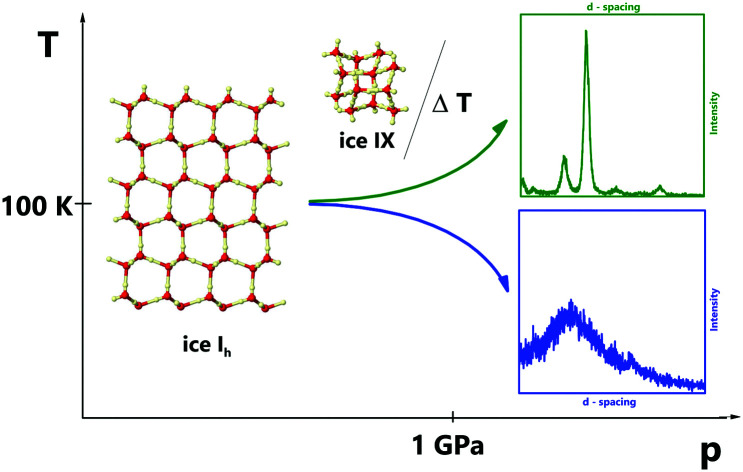

Liquid water is anomalous, especially in its supercooled state. One of the key properties distinguishing it from most other liquids is “polyamorphism”.^1^ Low- (LDA) and high-density amorphous ice (HDA) may be regarded as glassy solids, turning into two distinct supercooled liquids above their glass-to-liquid transition temperatures *T*_g_.^2–6^ At ambient pressure the two *T*_g_s for LDA and HDA are 136 K and 116 K, respectively.^7^ In the pressure range up to 1.6 GPa they vary between 110 and 160 K.^8^ One of the key questions is whether the amorphous ices are stable enough to turn from non-equilibrium states into equilibrated liquids before they crystallise. This necessitates that activation barriers against crystallisation are sufficiently high, *i.e.*, thermal fluctuations at 110–160 K cause them to turn into ultraviscous liquids, without crystallisation. In their recent work Tulk *et al.* negate this possibility by claiming that crystallisation to ice IX rather than formation of HDA takes place for slow compression experiments of hexagonal ice I_h_ at 100 K.^9^ Specifically, they compress ice I_h_ at 100 K at two different rates. At the higher rate (∼50 MPa min^−1^) they observe the well-known pressure-induced amorphisation (PIA) I_h_ → HDA near 1 GPa,^1,10^ followed by crystallisation of HDA to ice VII near 3 GPa. By using a slower, stepwise compression they obtain a different transition sequence: I_h_ → IX at 0.3 GPa, followed by IX → XV above 1 GPa, and at 3 GPa ice VIII forms. They interpret HDA formation as a “kinetically inhibited result of interrupted crystallisation of I_h_”, *i.e.*, HDA seems to be a transition state between ice I_h_ and ice XV that is not thermodynamically connected with high-density liquid water.

We here take a stand on the controversy emerging from these findings, where they challenge the idea that HDA is a glassy form thermodynamically related to the liquid. Their results contradict the results obtained in the pioneering work by Mishima *et al.*^1,10^ that were later confirmed by many groups, including Klotz *et al.*,^11^ Strässle *et al.*,^12^ Gromnitskaya *et al.*^13^ and our own group.^14^ While failing to reproduce the results in these literature studies, the results of Tulk *et al.* are quite similar to results in our own group obtained at much higher compression temperature.^15,16^ Specifically, we have shown in M. Bauer *et al.* that compression rate as well as compression temperature are of crucial importance to determine the outcome of compression experiments of ice I_h_.^15,16^ In that work we have shown that ice III/IX forms as the major product quite easily at 170 K, but ice II predominates at higher temperature. Faster compression favours the metastable form ice III/IX, and slower compression favours the thermodynamically stable form ice II. In order to obtain thermodynamically stable, pure ice II upon compression of ice I_h_, the compression needs to be slower than 2 MPa min^−1^ at 170 K, but can be as fast as 100 MPa min^−1^ at 220 K (see Fig. 4 in ref. 16). In other words, metastable ice III/IX is favoured at lower temperatures and higher compression rates over stable ice II. Exactly the same principle applies for the formation of HDA *vs.* ice III/IX, where in this case HDA is metastable with respect to ice III/IX, which itself is metastable with respect to stable ice II. Throughout this manuscript, we define the term “metastable” in the sense that relaxation times are shorter than transformation times by orders of magnitude. This implies that equilibration is achieved prior to transformation. Based on the dielectric relaxation times measured by Lemke *et al.*^17^ this criterion is fulfilled for HDA, where dielectric relaxation times are approximately ten thousand times shorter than the polyamorphic transition to LDA. The metastable HDA only forms at lower temperature whereas ice III/IX (or even ice II) forms at higher temperature. The compression rate dependence and the phases emerging found by Tulk *et al.*^9^ are exactly the same as that reported by us in ref. 15 and 16, but at 100 K instead of 170 K.

R. Bauer *et al.*^18^ repeated the experiment by Tulk *et al.*^9^ at a different high-pressure neutron diffraction setup (J-PARC MLF), where they find that HDA indeed does form at 100 K in slow compression experiments, not ice III/IX. In spite of the contradicting results, R. Bauer *et al.* come up with an interpretation similar to the one of Tulk *et al.*, *i.e.*, formation of HDA is the result of an incomplete transition between two crystalline forms of ice, that is, ice I_h_ and ice VI/XV.^18^ In order to clarify what the experimental findings actually are and how to understand the nature of HDA we conducted slow compression experiments following their protocol, but with direct measurement of pressure and temperature in our piston-cylinder setup and using powder X-ray diffraction for sample characterisation. This extends our earlier work done at 170–230 K^15,16^ to lower temperature. Furthermore, we have purposefully contaminated ice I_h_ samples with ice III/IX to study the impact of these impurities on the outcome of the slow compression experiment.

On the methodological side, the slow compression experiments were done following the procedures established over the last 20 years in our group.^19,20^ 400 μL of ultrapure liquid H_2_O were pipetted into a precooled container made of ∼0.7 g indium foil. After crystallisation of water to hexagonal ice, the sample container was inserted into the 8 mm bore of our custom-made high-pressure piston cylinder setup that is pressurised using a commercial “universal material testing machine” (Zwick, model BZ100/TL3S). The high-pressure cell was cooled by liquid nitrogen flowing through a copper loop around the cylinder. Heating elements as well as a Pt-100 temperature sensor were inserted in additional bores in the cell, 1 cm from the sample position (see Fig. 3.1 in ref. 21 or Fig. 10 in ref. 22 for a schematic drawing of our cell/setup). This apparatus enables the simultaneous detection and control of volume change, temperature and pressure in real time. The samples were pre-compressed (except for the sample compressed at 4 MPa min^−1^ shown in ESI,† Fig. S1a and S2a) to 0.4 GPa at 100 MPa min^−1^ and 77 K in order to remove air trapped within the ice. Then the sample was decompressed to 0.02 GPa at 20 MPa min^−1^ and heated to the desired temperature. Ice IX impurities were introduced on purpose into some ice I_h_ samples as follows: hexagonal ice was isobarically heated to 170 K at 0.02 GPa and then compressed at 100 MPa min^−1^. At ∼0.32 GPa, in the process of transformation to IX, the sample was quenched by pouring liquid nitrogen over the cell. Depending on the progress of ice IX formation until quenching, the amount of ice IX impurity in a mixed sample could be up to ∼50%. After reaching the end pressure at a certain temperature, samples were quench-recovered and analysed using X-ray diffraction (Cu-K_α_) at temperatures <80 K and subambient pressure.

Fig. 1a shows volume curves upon compression of ice I_h_ at 100 K at ∼4 MPa min^−1^, which corresponds to the linear equivalent of the stepwise compression profile used by Tulk *et al.*^9^ These curves exhibit linear densification up to 0.94 GPa when a steplike densification of about 20% occurs. X-Ray diffractograms (see Fig. 1b) reveal that ice I_h_ is stable up to 0.8 GPa, whereas Tulk *et al.* have already observed ice IX at 0.3 GPa. At 1.0 GPa, first signs of the HDA halo peak (maximum at *d* = 2.9 Å) become apparent (emerging halo peak marked by vertical arrow), and at 1.4 GPa only the halo peak is observed, with no signs of any Bragg peaks. That is, pressure-induced amorphisation is complete and HDA easily survives for hours at *p* > 1 GPa at 100 K, whereas ice IX does not appear at all.

**Fig. 1:**
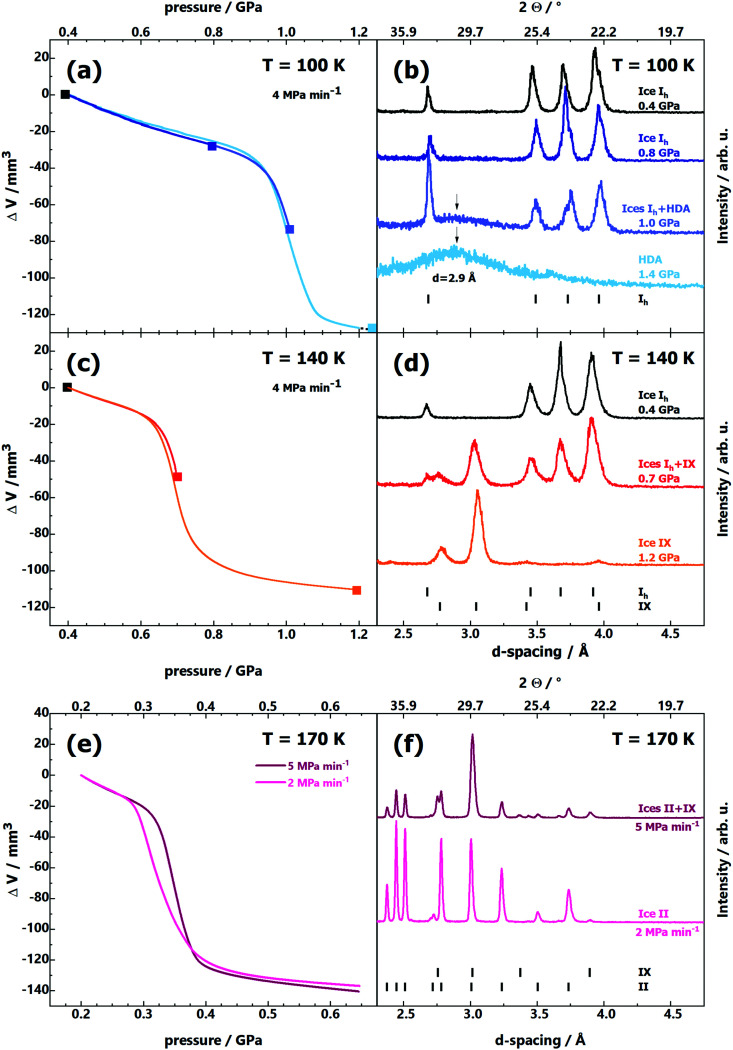
(a, c and e) Volume curves Δ*V*(*p*) obtained upon compression of pure hexagonal ice I_h_ at (a) 100 K, (c) 140 K and (e) 170 K and a constant compression rate of (a and c) 4 MPa min^−1^ and (e) 2 and 5 MPa min^−1^. (b, d and f) Series of powder X-ray diffractograms of quench-recovered samples after compression at (b) 100 K, (d) 140 K, (f) 170 K and 4 MPa min^−1^. Samples were recovered from the pressures marked by filled squares in (a and c), where the diffractograms are plotted in the same colour as the square. Diffraction angles at top *x*-axis correspond to Cu-K_α_ radiation.

In order to mimic the experiment by Tulk *et al.* even more closely, we have also used a stepwise compression protocol at 100 K. For this purpose, pressure was increased using a step-profile, using 50 MPa min^−1^ for each step, and maintaining the plateaus for 1 hour (see incrementally coloured Δ*V*(*p*) line in ESI,† Fig. S1a). No matter whether we use the linear ramp or the stepwise protocol (see ESI,† Fig. S1b), HDA is produced, not crystalline ices. X-Ray diffractograms of the resulting samples feature the broad halo peak typical of HDA, but no sharp Bragg peaks typical of crystalline ice (shown as insets in ESI,† Fig. S1a). Even at half the compression rate used by Tulk *et al.* (2 MPa min^−1^) we do not find any signs of crystalline ice IX at 100 K. The respective (a) Δ*V*(*p*) and (b) *p(t)* lines (red) are compared with the ones of an experiment conducted at 4 MPa min^−1^ (black) in ESI,† Fig. S2. Again, the X-ray diffractograms (inset in ESI,† Fig. S2a) exhibit the formation of only HDA (with a minor amount of ice I_h_). Even though this experiment takes about 10 hours, HDA survives. There are no signs of any crystalline high-pressure ice phases after such a long experiment. This demonstrates the longevity of HDA and implies that at 100 K the activation barrier is high enough and the prefactor small enough so that none of the attempts to cross the barrier for HDA crystallisation is successful. There might be an even slower compression experiment that allows formation of metastable ice III/IX (or even stable ice II) at 100 K from ice I_h_. However, such an experiment would certainly exceed the limit of available time resources for a PhD thesis. We surmise that the ice I_h_ sample would need to be kept for years at 100 K to actually reach conversion to an ice that is thermodynamically more stable than HDA. Put in other words, HDA is an ice form of longevity at 100 K, maybe even an infinitely stable phase.

The situation changes at 140 K (see Fig. 1c and d): instead of amorphisation at 1.0 GPa we observe crystallisation to ice IX at 0.65 GPa (Fig. 1c). The steplike densification occurs at much lower pressure at 140 K. In the middle of the densification, at 0.7 GPa (red Δ*V*(*p*) line in Fig. 1c), we observe Bragg peaks both from ice I_h_ and ice IX, indicating that the transition is in progress, but incomplete at 0.7 GPa (see red middle diffractogram in Fig. 1d). At 1.2 GPa, far above the offset of densification (orange Δ*V*(*p*) line in Fig. 1c), the diffractograms no longer show any signs of a broad halo peak, but only ice IX Bragg peaks (see orange bottom diffractogram in Fig. 1d). That is, at this elevated temperature we observe the transition I_h_ → IX at 4 MPa min^−1^. Yet, the onset pressure of the transformation (0.65 GPa) is significantly higher compared to the one reported by Tulk *et al.* (0.3 GPa). Finally, at 170 K (see Fig. 1e and f) we observe a transition starting at ∼0.3 GPa. For a compression rate of 5 MPa min^−1^ (purple line in Fig. 1e) stable ice II and some metastable ice IX form (see upper diffractogram in Fig. 1f). Only for a rate of 2 MPa min^−1^ (pink line in Fig. 1e) at 170 K thermodynamics ultimately win, and the stable ice II is formed (see lower diffractogram in Fig. 1f). Again, our results at 170 K are quite similar to the ones by Tulk *et al.* at 100 K, *i.e.*, there is a huge offset of 70 K.

That is, sample temperature and its measurement could be one source of the discrepancy. However, a stable temperature gradient of ∼70 K between sample and sensor, unnoticed by Tulk *et al.* seems unrealistic. In the interest of full transparency, we here show our temperature sensor data for all the experiments considered in the present study in ESI,† Fig. S3. In general the temperature remains constant to better than ±0.3 K for many hours. At some points in the experiment the flow through the cooling loops suddenly changed (*e.g.*, through dirt or ice in the lines). In such situations the temperature fluctuations increase, and a bit larger deviations from the set temperature appear for a few minutes, after which the set temperature is reached again (*e.g.*, yellow line for set temperature 130 K in ESI,† Fig. S3b). Since the temperature sensor is very close to the sample and remains constant for many hours we regard the sensor temperature to exactly reflect the sample temperature, so that we trust our temperatures to better than ±1 K.

In order to rule out, that an isotope effect (H_2_O ↔ D_2_O) is at the origin of the difference between the results obtained by Tulk *et al.* and ourselves, we conducted a control experiment compressing ∼400 μL D_2_O ice I_h_ at 100 K at a rate of 4 MPa min^−1^ (see ESI,† Fig. S4). As expected, we observed the same transformation as for H_2_O, *i.e.*, pressure-induced amorphisation. The main difference is a slightly higher onset pressure for the transformation. Such small isotope effects are typical for phase-transformations, in which the oxygen-lattice is changing, such as pressure-amorphisation.

An actual source for the deviating results of Tulk *et al.* might be the presence of nano-sized ice IX seeds in their ice I_h_ samples, too small to be detected by neutron diffraction. They mention precautions to avoid contamination with other ice phases upon closing their cell.^9^ Yet, slight fluctuations of gas pressure during the increase of load on the Paris–Edinburgh press could induce abrupt changes of the compression rate during the experiment, causing uncontrolled shock-wave heating.^23^ Such a sudden heating event, taking place within a millisecond, could remain unnoticed. Shock-wave heating is avoided in our piston-cylinder experiments by the use of indium as a low-temperature lubricant and controlled build-up of pressure from the first moment on. This concept goes back to the work of Mishima.^1^ In the absence of indium a loud bang and a massive drop in pressure indicate the release of the shock-wave. Compression experiments of ice I_h_ inflicted with shock-wave heating were conducted by Kohl *et al.*^24^ They have shown that such shock-waves may raise the sample temperature by up to 100 K, causing formation of ice XII, provided some initial HDA has formed through PIA at >0.9 GPa. Shock-wave heating of ice I_h_ at lower pressure might produce other ice phases, *e.g.*, ice IX at <0.2 GPa.

For this reason, we studied the influence of ice IX contamination by slowly compressing mixtures of I_h_/IX at 100–140 K (see Fig. 2). Depending on temperature, the volume curves in Fig. 2a and the X-ray diffractograms in Fig. 2b show three distinct outcomes: below 120 K, the Δ*V*(*p*) curves reveal only a single I_h_ → HDA densification step at ∼1 GPa (purple and blue curves in Fig. 2a). The presence of ice IX contamination barely affects pressure-amorphisation; compression curves are almost identical to the pure ice I_h_ case (see Fig. 1a). There is only a very small change of onset pressure when comparing compression of pure ice I_h_ and an ice I_h_/IX mixture (see arrow directed upwards in Fig. 2c at 100 K). At 120–130 K both transitions, I_h_ → IX and I_h_ → HDA, occur well separated in terms of pressure. The compression curves (light-blue, green and yellow in Fig. 2a) show two kinks, one near 0.4 GPa, the other near 0.9 GPa. The first kink indicates the onset of ice IX formation, the second kink indicates formation of HDA from the remainder of ice I_h_. That is, there is a competition between amorphisation and polymorphic transformation under these conditions. The horizontal dotted lines in Fig. 2a indicate the volume change incurred through ice IX formation from ice I_h_ (Δ*V*_Ih→IX_) as well as the volume change incurred through HDA formation (Δ*V*_Ih→HDA_). Fig. 2d shows that the HDA-fraction is 100% below 120 K and drops to about 50% at 130 K. The fraction of ice IX increases with temperature, at the expense of HDA. This is also evident in the X-ray diffractograms in Fig. 2b (coloured traces in the middle). It is clearly seen that the HDA halo marked by an arrow becomes less and less pronounced with higher compression temperature. Above 130 K there is only ice IX, but no HDA. At 135 K and 140 K (orange and red curves in Fig. 2a) there is a single densification step again, where the transition is I_h_ → IX now. When comparing the onset pressure of ice IX growth at 140 K between compression of pure ice I_h_ (Fig. 1e) and compression of ice I_h_/IX (Fig. 2a, red curve) a marked difference is evident. Ice IX impurities act as seeds for ice IX formation and lower the onset pressure from 0.65 GPa to 0.40 GPa at 140 K (downward-arrow in Fig. 2c). This has implications for our understanding of the issues in the experiment by Tulk *et al.* on PIA of ice I_h_: a short-term temperature gradient between sensor and sample of about 30–40 K and some nano-scaled ice IX contamination (undetected by neutron diffraction) as a result of shock-wave heating could explain the incompatibility with the experiments in other groups.

**Fig. 2:**
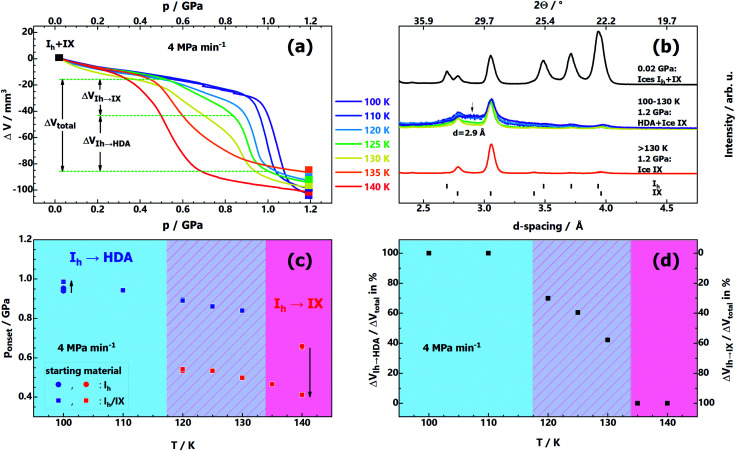
(a) Volume curves Δ*V*(*p*) obtained upon compression of mixtures of ices I_h_/IX at temperatures 100–140 K and a constant compression rate of 4 MPa min^−1^. (b) Powder X-ray diffractograms of quench-recovered samples after compression to 1.2 GPa at 4 GPa min^−1^. The top diffractogram represents the starting material for this series of experiments, that is, mixtures of I_h_/IX. Diffraction angles at top *x*-axis correspond to Cu-K_α_ radiation. (c) Onset pressures for the transitions I_h_ → IX and I_h_ → HDA in samples of I_h_/IX as a function of temperature *p*_onset_(*T*). The reproducibility is better than 0.01 GPa. Onset pressures of the transitions of pure ice I_h_ at 100 K and 140 K (extracted from Fig. 1a and c) are shown (blue and red circles) for comparison. The onset pressures are defined as the intersection point between two linear functions fitting the linear section of the Δ*V*(*p*) curves in (a) just before and during the transition. (d) Evaluation of the densification step of the volume curves Δ*V*(*p*) in (a). At low and high temperatures, either I_h_ → HDA or I_h_ → IX is operative. The total change of volume Δ*V*_total_ in the intermediate temperature region is composed of the volume changes of both transition modes, Δ*V*_Ih→IX_ and Δ*V*_Ih→HDA_.

## Discussion and Conclusions

**Fig. 3:**
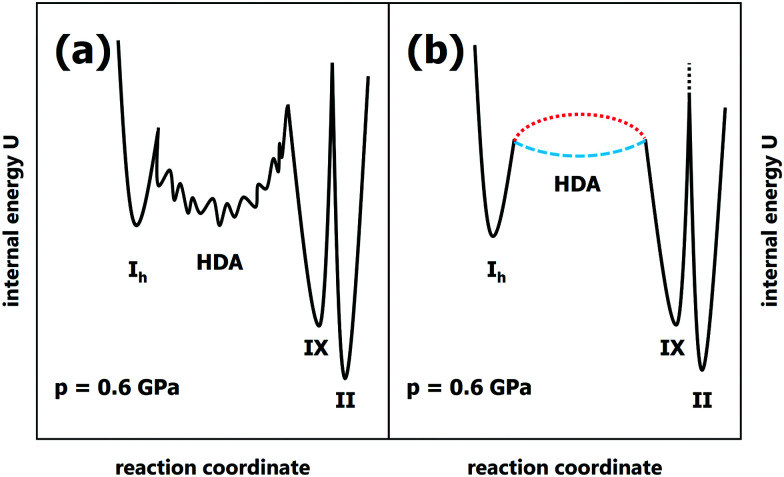
Internal energy sketches for the scenarios proposed by (a) us and (b) Tulk *et al*.^9^

Our data obtained for slow compression of ice I_h_ at 100–170 K are consistent with most earlier literature studies, but inconsistent with the findings reported by Tulk *et al*.^9^ We rationalise the discrepancies with shock-wave heating causing a short-term temperature gradient between their sample and sensor and contamination with nano-sized impurities of ice IX. Contrasting the interpretations of Tulk *et al.*^9^ and R. Bauer *et al.*,^18^ we show that HDA is not an unstable, derailed or frustrated state incurred in the conversion from ice I_h_ to ice VI/XV, but long-lived at 100 K. In fact, we have never ever seen conversion of HDA to crystalline ices in all our piston-cylinder experiments at 100 K. The inability to detect HDA in their slow experiments at 100 K is in contradiction with lifetimes deduced in earlier work: HDA survives for at least 3 hours at 0.2 GPa even at 130 K^13^ and resists crystallisation up to ∼155 K at 0.3 GPa.^25^ Even above its glass-to-liquid transition temperature HDA is stable over many hours.^26^ Lemke *et al.* have quantified that about 10.000 relaxation times have to pass before HDA leaves its potential energy megabasin at ∼120 K (see Fig. 6 in ref. 17). Fig. 3a summarises our findings in terms of a potential energy sketch that shows HDA to be a deep basin on the landscape, metastable with respect to both ice III/IX and ice II. In this basin equilibration may take place, where large barriers prevent crystallisation. The opposing view implied in the work by Tulk *et al.*^9^ and advocated by R. Bauer *et al.*^18^ against their own experimental evidence is depicted in Fig. 3b. In this opposing view HDA represents a very shallow basin (dashed blue line) or even a transition state (dotted red line) that cannot be isolated and kept as a kinetically stable state.

This view is inconsistent with all experimental findings on PIA of ice I_h_, except for the work from Tulk *et al.*, which we suggest to be afflicted with issues of temperature gradients and ice IX contamination based on our findings. This sets the experimental record straight and confirms earlier work on pressure-induced amorphisation of ice I_h_. Furthermore, we point out that Tulk *et al.* themselves observe a metastable ice, namely ice IX, rather than the stable ice II at 0.3 GPa. This means that they do not reach the thermodynamic limit and the equilibrium form of ice, but instead an ice that is kinetically stabilised. In their case the barrier of polymorphic conversion from ice IX to ice II is high enough to prevent the most stable ice form (see transition state between ice IX and ice II in Fig. 3, marked by black-dotted line). Rather than observing stable ice II they observe that metastable ice IX persists. Our earlier work, M. Bauer *et al.*,^15^ shows that a temperature of 230 K or above is necessary to overcome this barrier for the polymorphic transition and to reach ice II in the slow compression limit. Just like ice IX at 0.3 GPa also HDA is kinetically stable at >1 GPa, where 140 K should not be exceeded to avoid HDA crystallisation on the time scale of hours. Finally, Tulk *et al.* have not accounted for the huge enhancement of HDA lifetime after its annealing above 1 GPa.^27,28^ Annealing removes nano-sized crystalline domains serving as growth sites for crystalline ice and thus enhances stability.^29^ What Tulk *et al.* produce by PIA at 100 K is referred to as unannealed HDA (uHDA),^27^ while only the much more stable expanded HDA (eHDA) was found to be directly related to ultraviscous water.^25^ This leads to the conclusion that the activation barriers for HDA crystallisation are significantly higher than claimed by Tulk *et al.*^9^ as well as R. Bauer *et al.*^18^ In fact, they are high enough to keep HDA stable above its glass-to-liquid transition temperature, so that high-density liquid water (HDL) may equilibrate instead of crystallisation to ice IV and ice XII. This has been demonstrated in the recent work of our group by Stern & Loerting^30^ as well as Handle & Loerting.^31^

While the recent experimental findings presented by R. Bauer *et al.*^18^ are consistent with the results presented in Fig. 1, there is a significant difference about the interpretation of the nature of HDA. They claim HDA to be a frustrated state between ice I_h_ and ice VI/XV, contrasting our claim above of HDA being a metastable phase related to the high-pressure liquid state. Similarly Shephard *et al.* claim HDA to be a derailed state along the ice I_h_ to ice IV conversion.^32^ The resistance of HDA against crystallisation for many hours at 120 K speaks against both ideas of HDA being a transient state in a polymorphic conversion. In slow heating experiments HDA even resists crystallisation up to 161 K.^30^ Also the conjecture by R. Bauer *et al.*^18^ that amorphisation represents the onset from a single H-bond network to an interpenetrating H-bond network defies experimental evidence. In reality, HDA always crystallises first to the single H-bond network ice IV and/or ice XII,^33,34^ but not directly to ice VI/XV with interpenetrating H-bond networks. In fact, the transition to interpenetrating networks does not require an amorphous intermediate, but directly takes place in the polymorphic ice IV → ice VI or XII → ice VI transition.

Taken together this experimental evidence allows regarding HDA/HDL as a “metastable phase” in the thermodynamic sense rather than a “transient state”, in spite of its glassy nature. Unlike “simple amorphous states”, metastable HDA/HDL can be reached through many different thermodynamic paths, where pressure-induced amorphisation studied in the present work is only one of them. Other ways include cooling of the pressurised liquid,^35^ temperature-induced amorphisation of ice,^36^ decompression-induced melting of high-pressure ice,^37^ or compression of hyperquenched water,^38^ to name just a few. This is only possible if HDA/HDL is describable as a deep basin (as sketched in Fig. 3a), where many paths lead to the same, local minimum on the surface.

## Author contributions

C. M. T. and T. L. conceived and designed the experiment, analysed the data and wrote the manuscript. C. M. T. and M. B. conducted the experiments.

## Conflicts of interest

There are no conflicts to declare.

## Supplementary Material

CP-024-D1CP03922A-s001
