# Supplementary material for: The impact of temperature and unwanted impurities on slow compression of ice
Source: Phys Chem Chem Phys. 2021 Dec 6;24(1):35–41. doi: 10.1039/d1cp03922a (PMC8694060; doi:10.1039/d1cp03922a)
Supplement: CP-024-D1CP03922A-s001 [file CP-024-D1CP03922A-s001.pdf]

Electronic Supplementary Material (ESI) for Physical Chemistry Chemical Physics.
This journal is © the Owner Societies 2021

The impact of temperature and unwanted impurities on slow compression of ice

Christina M. Tonauer, Marion Bauer[a], Thomas Loerting*

Institute of Physical Chemistry, University of Innsbruck, Innrain 52c, A-6020 Innsbruck, Austria

a *current address: Sandoz GmbH, Biochemiestr. 10, A-6250 Kundl, Austria*

* e-mail: thomas.loerting@uibk.ac.at

SUPPLEMENTARY FIGURES

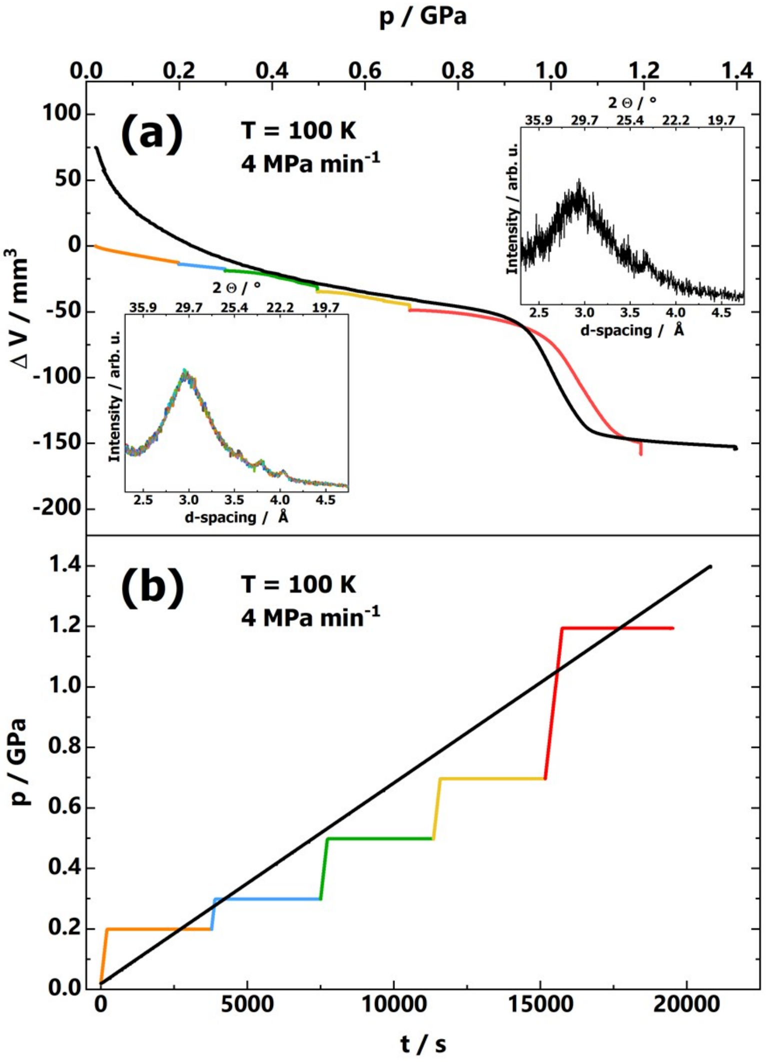

Supplementary Fig. 1:

(a) Comparison of volume change *ΔV(p)* upon a stepwise increase in pressure (coloured line) and a continuous increase (black line) of 4 MPa min^{-1} at 100 K. The sample used for the continuous compression experiment was not pre-compressed. Therefore, the non-linear densification until ~ 0.4 GPa is based on the release of air from the sample. The respective X-ray diffractograms (bottom left inset: stepwise increase, top right inset: continuous increase) reveal that both compression protocols yield HDA. Diffraction angles at top x-axis correspond to Cu-Kα radiation. **(b)** Compression protocol of the compression experiments shown in (a)**.**

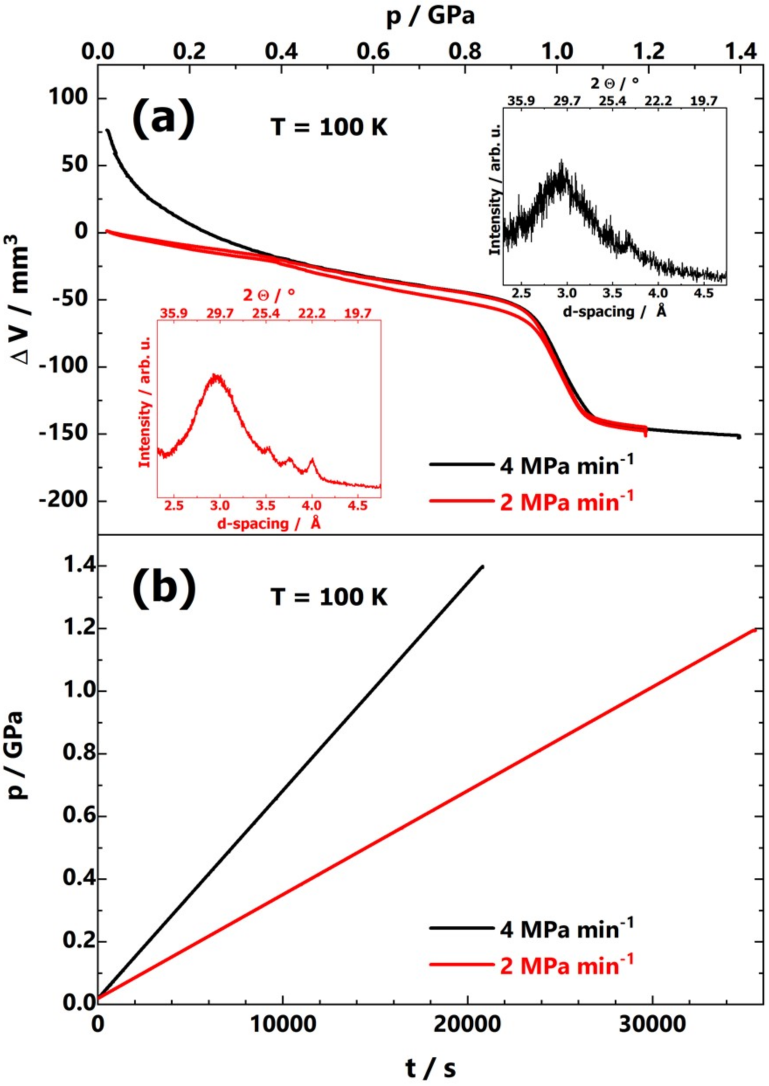

Supplementary Fig. 2:

(a) Comparison of volume change *ΔV(p)* for a constant compression rate of 2 MPa min^{-1} (red) and 4 MPa min^{-1} (black) at 100 K. The sample used for the experiment at 4 MPa min^{-1} was not pre-compressed. Therefore, the non-linear densification until ~ 0.4 GPa is based on the release of air from the sample. In both cases, the respective X-ray diffractograms (bottom left: 2 MPa min^{-1}, top right: 4 MPa min^{-1}) reveal the formation of HDA. Diffraction angles at top x-axis correspond to Cu-Kα radiation. **(b)** Compression protocols for experiments in (a).

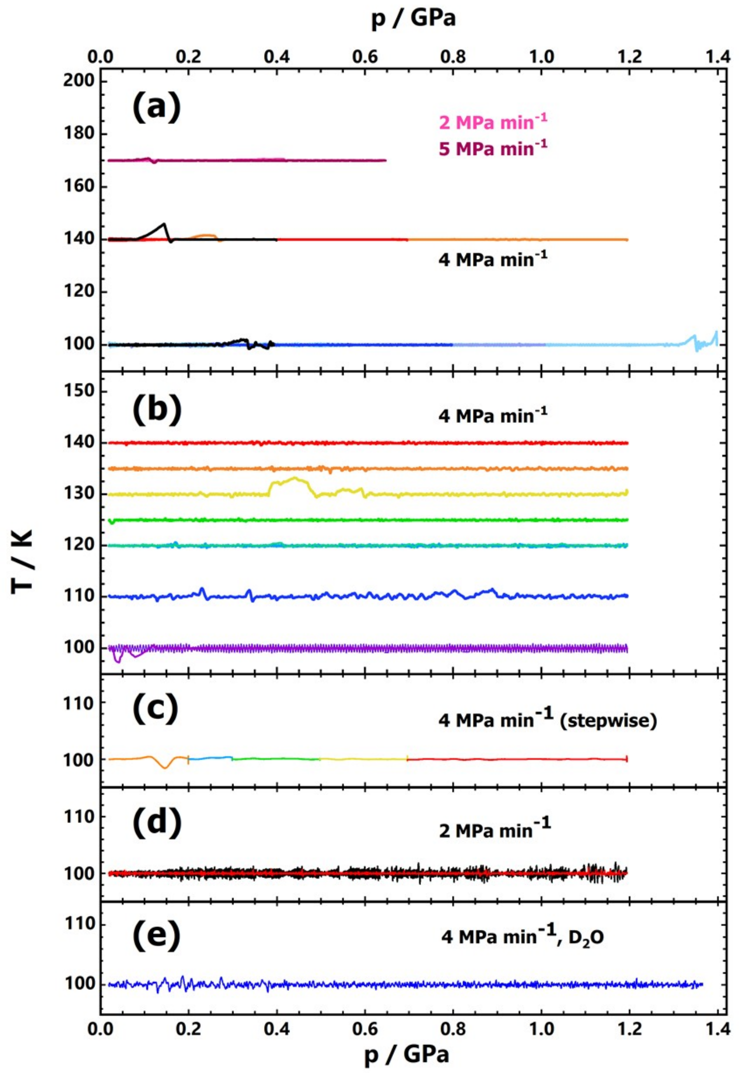

Supplementary Fig. 3: Measured Pt-100 temperatures for the (quasi)-isothermal slow compression experiments of **(a)** pure hexagonal ice shown in Fig. 1a, 1c and 1e and of **(b)** mixtures of I_h/IX shown in Fig. 2a. **(c)** *T(p)* lines of the stepwise compression experiment at 4 MPa min^{-1} depicted in Supplementary Fig. 1a, **(d)** of two experiments at a constant rate of 2 MPa min^{-1} shown in Supplementary Fig. 2a and **(e)** of a slow compression experiment at a constant rate of 4 MPa min^{-1} using D_2O, shown in Supplementary Fig. 4.

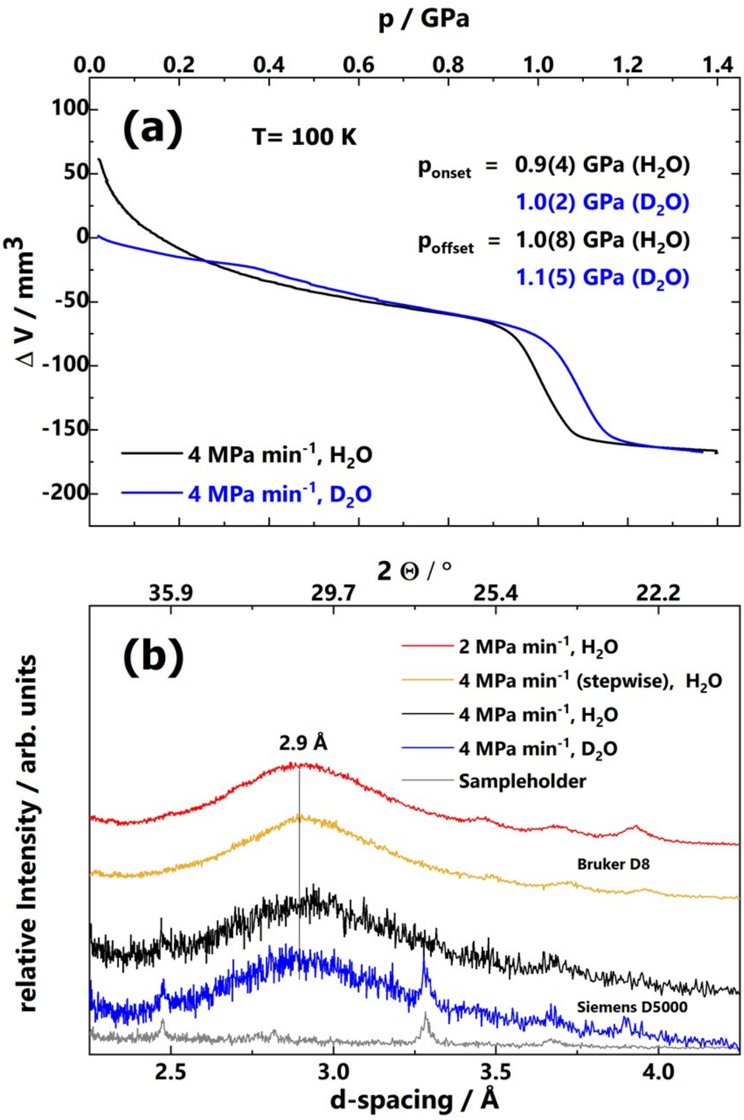

Supplementary Fig. 4: (a) Comparison of volume change *ΔV(p)* upon compression at 4 MPa min^{-1} at 100 K for a H_2O sample (black) and a D_2O sample (blue). The H_2O sample was not pre-compressed. Therefore, the non-linear densification until ~ 0.4 GPa is based on the release of air from the sample. Onset and offset pressures (p_{onset}, p_{offset}) of the steplike transition are defined by the intersection points of tangents along the *ΔV(p)* curve ahead of the transition, at the transition and after the transition, respectively. **(b)** Comparison of powder x-ray diffractograms measured at ~ 77 K (depicted with an offset along the y-axis). Diffraction angles at top x-axis correspond to Cu-Kα radiation. The top two diffractograms were measured using the *Bruker D8* powder diffractometer, the others were measured on a *Siemens D5000*. Independent of the compression rate and the isotope (H_2O or D_2O), slow compression of ice I_h at 100 K results in formation of HDA (halo peak maximum at 2.9 Å) and no crystalline forms of ice.
